# Real-world COVID-19 vaccine effectiveness against the Omicron BA.2 variant in a SARS-CoV-2 infection-naive population

**DOI:** 10.1038/s41591-023-02219-5

**Published:** 2023-01-18

**Authors:** Jonathan J. Lau, Samuel M. S. Cheng, Kathy Leung, Cheuk Kwong Lee, Asmaa Hachim, Leo C. H. Tsang, Kenny W. H. Yam, Sara Chaothai, Kelvin K. H. Kwan, Zacary Y. H. Chai, Tiffany H. K. Lo, Masashi Mori, Chao Wu, Sophie A. Valkenburg, Gaya K. Amarasinghe, Eric H. Y. Lau, David S. C. Hui, Gabriel M. Leung, Malik Peiris, Joseph T. Wu

**Affiliations:** 1 WHO Collaborating Centre for Infectious Disease Epidemiology and Control, School of Public Health, Li Ka Shing Faculty of Medicine, The University of Hong Kong, Hong Kong SAR, China; 2 Laboratory of Data Discovery for Health (D24H), Hong Kong SAR, China; 3 School of Public Health, Li Ka Shing Faculty of Medicine, The University of Hong Kong, Hong Kong SAR, China; 4 The University of Hong Kong – Shenzhen Hospital, Shenzhen, China; 5 Hong Kong Red Cross Blood Transfusion Service, Hong Kong SAR, People’s Republic of China; 6 HKU-Pasteur Research Pole, Li Ka Shing Faculty of Medicine, The University of Hong Kong, Hong Kong SAR, China; 7 Research Institute for Bioresources and Biotechnology, Ishikawa Prefectural University, Nonoichi, Japan; 8 Department of Pathology and Immunology, Washington University School of Medicine at St. Louis, St. Louis, MO USA; 9 Department of Microbiology and Immunology, Peter Doherty Institute for Infection and Immunity, University of Melbourne, Melbourne, Victoria Australia; 10 Department of Medicine and Therapeutics and Stanley Ho Centre for Emerging Infectious Diseases, Faculty of Medicine, Chinese University of Hong Kong, Hong Kong SAR, China; 11 Centre for Immunology and Infection, Hong Kong SAR, China

**Keywords:** Viral infection, Epidemiology, SARS-CoV-2, Vaccines

## Abstract

The SARS-CoV-2 Omicron variant has demonstrated enhanced transmissibility and escape of vaccine-derived immunity. Although first-generation vaccines remain effective against severe disease and death, robust evidence on vaccine effectiveness (VE) against all Omicron infections, irrespective of symptoms, remains sparse. We used a community-wide serosurvey with 5,310 subjects to estimate how vaccination histories modulated risk of infection in infection-naive Hong Kong during a large wave of Omicron BA.2 epidemic in January–July 2022. We estimated that Omicron infected 45% (41–48%) of the local population. Three and four doses of BNT162b2 or CoronaVac were effective against Omicron infection 7 days after vaccination (VE of 48% (95% credible interval 34–64%) and 69% (46–98%) for three and four doses of BNT162b2, respectively; VE of 30% (1–66%) and 56% (6–97%) for three and four doses of CoronaVac, respectively). At 100 days after immunization, VE waned to 26% (7–41%) and 35% (10–71%) for three and four doses of BNT162b2, and to 6% (0–29%) and 11% (0–54%) for three and four doses of CoronaVac. The rapid waning of VE against infection conferred by first-generation vaccines and an increasingly complex viral evolutionary landscape highlight the necessity for rapidly deploying updated vaccines followed by vigilant monitoring of VE.

## Abstract (web summary)

An analysis of sera samples collected between January and July of 2022 in Hong Kong shows that the effectiveness of both the BNT162b2 and CoronaVac COVID-19 vaccines against SARS-CoV-2 Omicron BA.2 variant infection waned rapidly after the third and fourth doses.

## Main

During 1 January to 31 July 2022, Hong Kong experienced an unprecedented fifth wave of COVID-19 infections driven predominantly by the Omicron BA.2 variant (B.1.1.529.2) with 1,341,363 reported cases (18.4% of the total population) and 9,290 deaths (0.7%)^1^. The fifth wave dwarfed the previous four waves in terms of cumulative infection attack rate (IAR), which was nearly zero before 2022 given Hong Kong’s then-successful ‘dynamic Zero-Covid’ strategy. Thus, population immunity to SARS-CoV-2 was almost entirely vaccine-derived when the fifth wave began. The messenger RNA vaccine Comirnaty (BNT162b2 mRNA, BioNTech/Fosun-Pharma) and the inactivated CoronaVac vaccine (Sinovac Life Sciences) have been available free of charge to Hong Kong residents aged 18 and above from 26 February 2021. Since then, eligibility to receive BNT162b2 or CoronaVac had been gradually extended to adolescents and children aged 6 months or above, and boosters to adolescents aged 12 years or above. Population uptake of at least two doses of either vaccine increased from 4.7 million (64% of the total population) by 1 January 2022 to 6.5 million (89%) by 31 July 2022 (ref. ^1^).

**Fig. 1:**
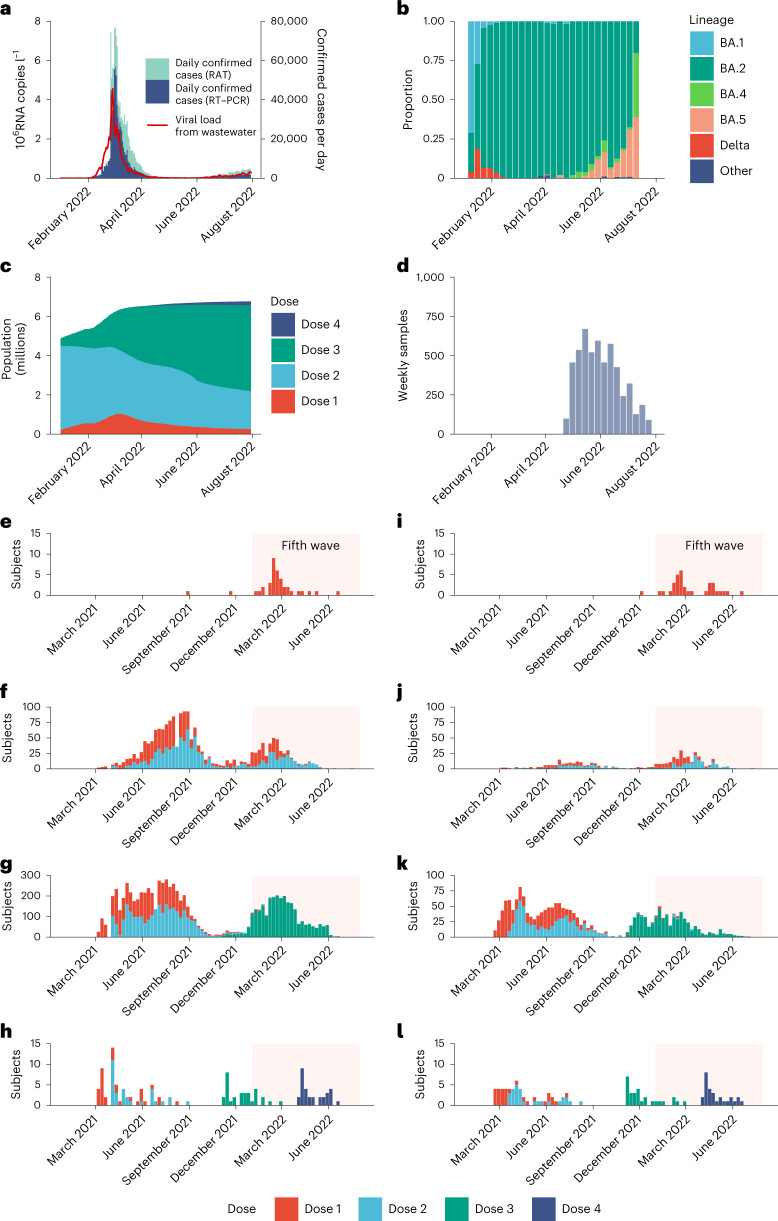
Daily COVID-19 confirmed cases, SARS-CoV-2 wastewater viral load, weekly proportion of SARS-CoV-2 lineages, population vaccination coverage, sera collection history and study subject vaccination history. **a**, Daily confirmed COVID-19 cases in Hong Kong stratified by cases confirmed by RT–PCR or RAT, superimposed against 2-day running geometric mean viral load per capita (in millions of copies of SARS-CoV-2 RNA l^−1^) detected in city-wide wastewater surveillance. **b**, Weekly proportion of SARS-CoV-2 lineages detected in Hong Kong via genome sequencing as uploaded to GISAID by 14 September 2022. **c**, Total population with one to four doses of BNT162b2 and/or CoronaVac in Hong Kong. **d**, Number of serum samples collected weekly. **e**–**h**, Vaccination history for study participants with one (**e**), two (**f**), three (**g**) and four (**h**) homologous doses of BNT162b2 at the time of sample collection. **i**–**l**, Vaccination history for study participants with one (**i**), two (**j**), three (**k)** and four (**l**) homologous doses of CoronaVac at the time of sample collection. The shaded areas in **e**–**l** correspond to the duration of the fifth wave from 1 January to 31 July 2022.

**Extended Data Fig. 1:**
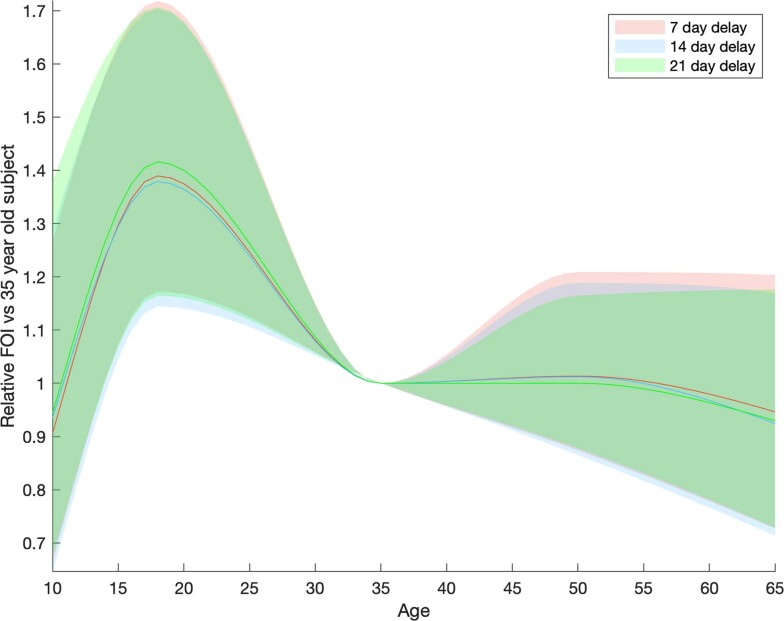
The effect of age on force of infection (FOI) (that is *f*(*a*) in the model). Individuals aged 35 years served as the reference group. The solid line indicates the posterior median, and the shaded regions indicate the 95% credible interval based on the fitted model, differentiated by assumption on delay to vaccine effectiveness taking effect.

We conducted a community-wide serosurvey to estimate: (1) age-specific IAR in the fifth wave; (2) age-specific population immunity in the fifth wave; and (3) VE against SARS-CoV-2 infection conferred by two, three and four homologous doses of BNT162b2 or CoronaVac for 100 days after each dose. Specifically, for each subject in our serosurvey, we estimated the probability of being infected by SARS-CoV-2 before study recruitment, given age, vaccination record and seropositivity of the serum sample (Methods). Correspondingly, we defined VE as the reduction in the probability of being infected by SARS-CoV-2 within the observation period, as conferred by the type and doses of vaccine received by the subject, relative to the probability of infection for an unvaccinated subject in the same period. Our estimates of VE and waning were specific to infection by Omicron BA.2 only because almost all COVID-19 infections in Hong Kong before our study period were BA.2. We assumed that: (1) daily age-specific force of infection (FOI) was proportional to daily viral load from city-wide wastewater surveillance (Fig. 1 and Extended Data Fig. 1), which has been shown to be a robust (normalized) proxy for disease prevalence over time^2–4^; and (2) one-dose vaccination provided no protection against infection and each successive homologous dose conferred greater VE that decayed exponentially over time at the same rate between doses of the same vaccine^5^.

## Results

Our serosurvey subjects included: (1) 5,173 healthy adult blood donors recruited from the Hong Kong Red Cross Blood Transfusion Service between 28 April and 30 July 2022; and (2) 137 children aged 18 months to 11 years randomly recruited from the community to participate in an independent polio seroepidemiology study (see Fig. 1d for sera collection history). Vaccination histories were available for 5,242 subjects (Fig. 1 and Table 1) from the Hong Kong Department of Health (98%) or self-report (2%). At the time of sample collection, 1,237 blood donors (24%) and 31 child subjects (23%) self-reported a previous infection.

**Table 1:** Characteristics of study participants, April 2022 to July 2022 (*n* = 5,310), excluding 67 participants with non-BNT162b2 or non-CoronaVac, or undetermined, vaccination history and one participant with undetermined age

	Overall (*n* = 5,242)		BNT162b2-exclusive (*n* = 3,759)		CoronaVac-exclusive (*n* = 906)		BNT162b2 and CoronaVac (*n* = 461)		Unvaccinated (*n* = 116)	
	Number of donors	Percentage of total	Number of donors	Percentage of subtotal	Number of donors	Percentage of subtotal	Number of donors	Percentage of subtotal	Number of donors	Percentage of subtotal
**Age group (years)**										
1–10	136	2.6	13	0.3	64	7.0	0	0.0	59	53.2
18–19	134	2.6	121	3.2	10	1.1	2	0.4	1	0.9
20–29	943	18.0	838	22.3	61	6.7	35	7.6	9	8.1
30–39	1,183	22.6	945	25.1	124	13.7	91	19.7	23	20.7
40–49	1,439	27.5	1,008	26.8	280	30.8	143	31.0	8	7.2
50–59	1,086	20.7	651	17.3	281	30.9	145	31.5	9	8.1
60–69	321	6.1	186	4.9	88	9.7	45	9.8	2	1.8
**Sex**										
Female	2,680	51.1	1,974	52.5	434	47.8	216	46.9	56	50.5
Male	2,548	48.6	1,787	47.5	473	52.1	245	53.1	43	38.7
Not-determinable	14	0.3	1	0.0	1	0.1	0	0.0	12	10.8
**Doses received**										
0	116	2.2	0	0.0	0	0.0	0	0.0	116	100
1	72	1.4	38	1.1	34	3.8	0	0.0	0	0.0
2	1,174	22.4	930	24.7	223	24.6	21	4.6	0	0.0
3	3,794	72.4	2,760	73.4	621	68.4	413	89.6	0	0.0
4	86	1.6	31	0.8	28	3.1	27	5.9	0	0.0

**Fig. 2:**
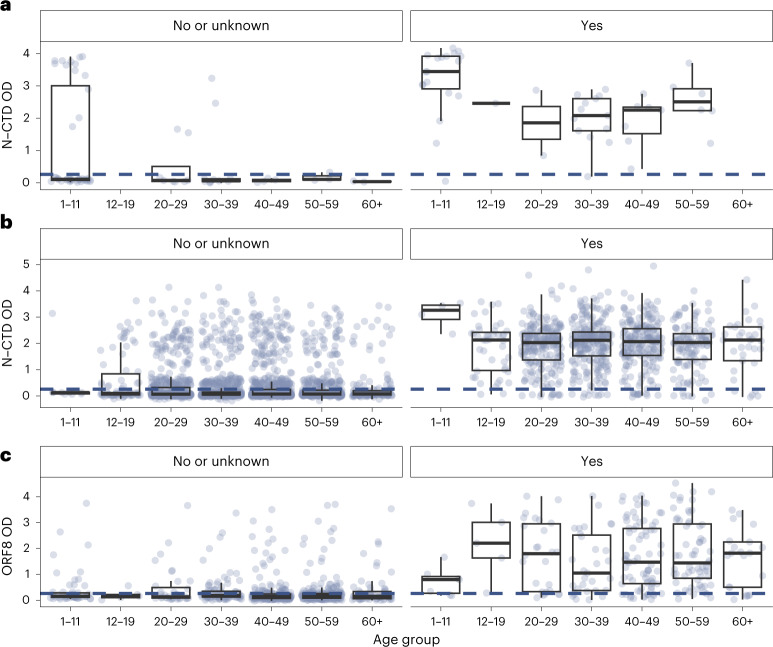
SARS-CoV-2 N-CTD and ORF8 antibody responses among study subjects by vaccination history, age and self-reported infection history. **a**–**c**, Unvaccinated (*n* = 116 subjects) (**a**), BNT homologously vaccinated (*n* = 3,759 subjects) (**b**) and CoronaVac homologously vaccinated (*n* = 906 subjects) (**c**). Panels on the left correspond to individuals with no self-reported infection history or who did not provide their infection history. Panels on the right correspond to individuals with self-reported infection history. The blue dotted line corresponds to the OD thresholds for seropositivity (N-CTD OD of 0.2583 for unvaccinated and BNT homologously vaccinated, and ORF8 OD of 0.33 for CoronaVac homologously vaccinated). Each dot represents one study subject. The center line of each box represents the median, box limits represent the interquartile range (IQR), and the whiskers represent the minimum and maximum observations greater and lesser than the IQR plus 1.5× IQR, respectively.

**Extended Data Fig. 2:**
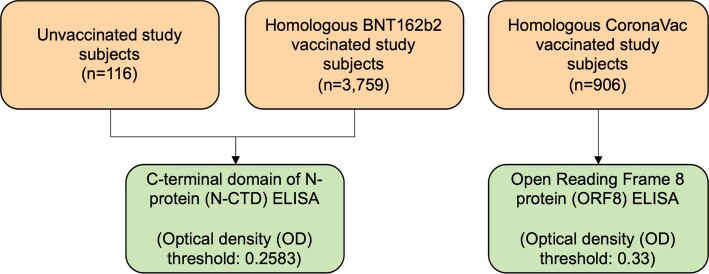
Use of ELISA assay to discriminate infection from vaccine immunity by vaccination cohort. This figure summarizes the in-house ELISA assays (green) we used to test for seropositivity amongst study subjects in different vaccination cohorts (orange). We did not test for seropositivity amongst heterologously vaccinated study subjects, study subjects who received vaccines other than BNT162b2 or CoronaVac, or study subjects with an un-determined vaccination history.

**Extended Data Fig. 3:**
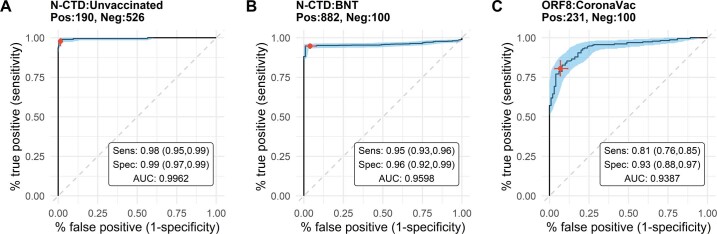
Description of controls, assay performance and Receiver Operating Curves (ROC) by ELISA assay type for detecting Omicron infection with in-house ELISA protocols. **(a)** N-CTD ELISA amongst unvaccinated controls, tested against 48 unvaccinated study subjects with self-reported infection history, 142 unvaccinated RT-PCR positive convalescent samples ranging from 30 to 401 days after onset of illness and 526 pre-pandemic negative controls; **(b)** N-CTD ELISA amongst controls who were homologously vaccinated with BNT162b2, tested against 881 BNT-vaccinated study subjects with self-reported infection history, 1 BNT-vaccinated RT-PCR positive convalescent sample collected 369 days after onset of illness and 50 (tested twice) non-infected BNT-vaccinated samples collected during 2020–2021, a period of minimal community transmission; and **(c)** ORF8 ELISA amongst controls who were homologously vaccinated with CoronaVac, tested against 231 CoronaVac-vaccinated study subjects with self-reported infection history and 100 non-infected CoronaVac-vaccinated samples collected during 2020–2021, a period of minimal community transmission. Shaded areas indicate 95% confidence region. Red dot and cross represent the median sensitivity and specificity and corresponding confidence intervals of the OD threshold jointly estimated via Gibbs Sampling as described in Methods. Pos: positive controls, Neg: negative controls, Sens: sensitivity, Spec: specificity, AUC: area under the curve.

We developed two in-house enzyme-linked immunosorbent assays (ELISA) detecting immunoglobulin (Ig)G antibodies to the C-terminal domain of the nucleocapsid (N) protein (N-CTD) and the Open Reading Frame 8 protein (ORF8) of SARS-CoV-2, respectively, modifying and validating previously reported methods^6,7^. We estimated that our N-CTD assay was more than 95% sensitive and 96% specific in detecting recent Omicron infection among unvaccinated individuals and homologous BNT162b2 vaccinees. Because the inactivated whole-virus vaccine CoronaVac elicits antibody to the N protein, the ORF8 assay was optimized specifically for discriminating between infection and vaccine-derived antibody in CoronaVac vaccinees. We estimated that our ORF8 assay was 81% sensitive and 93% specific in detecting recent Omicron infection among homologous CoronaVac vaccinees. See Extended Data Fig. 2 for a mapping of vaccination cohort by assay. See Methods, Fig. 2 and Extended Data Fig. 3 for further details on assay workflow, performance and output. To our knowledge, our ORF8 assay is the first serological test that could effectively detect and discriminate recent SARS-CoV-2 infection from vaccination among CoronaVac vaccinees.

Assuming VE took full effect 7 days after vaccination, we estimated: (1) VE for the second, third or fourth doses of BNT162b2 were 13% (95% credible interval: 2–39%), 48% (34–64%) and 69% (46–98%) 7 days following immunization, respectively, waning to 7% (1–21%), 26% (7–41%) and 35% (10–71%) 100 days after immunization; and (2) VE for the second, third or fourth dose of the CoronaVac vaccine were 5% (0–27%), 30% (1–66%) and 56% (6–97%) 7 days following immunization, respectively, waning to 1% (0–11%), 6% (0–29%) and 11% (0–54%) 100 days after immunization.

**Fig. 3:**
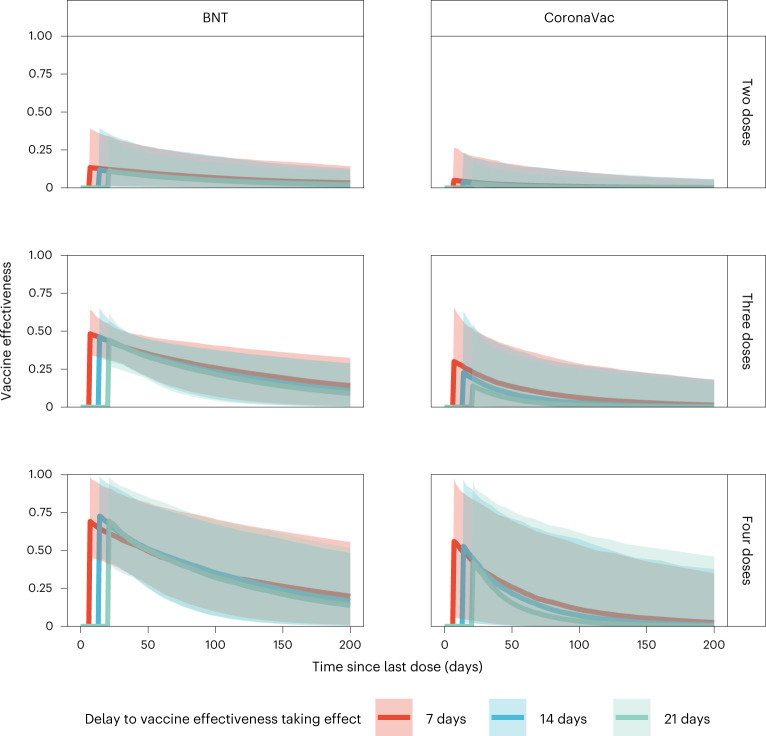
Estimated VE. VE at 0 to 200 days from receipt of last dose. VE is presented separately over time for two, three or four homologous doses of BNT162b2 (BNT) or CoronaVac. The lines indicate posterior medians and shaded bars indicate 95% credible intervals based on the fitted model.

**Extended Data Fig. 4:**
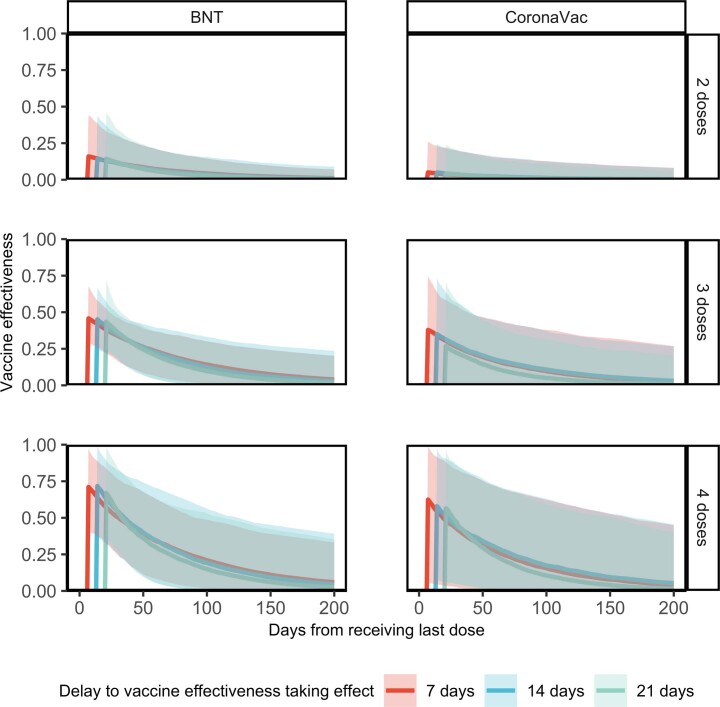
Vaccine effectiveness over time only including samples collected on or before 15 June 2022. VE at zero to 200 days from receipt of last dose. VE is presented separately over time for two, three or four homologous doses of BNT162b2 (BNT) or CoronaVac. The lines indicate posterior medians and shaded bars indicate 95% credible intervals based on the fitted model.

Studies conducted during Omicron BA.1/BA.2 dominance and involving three adult doses^8^, four adult doses^9^ or two adolescent (12–17 years old) doses^10^ of BNT162b2 vaccination indicated full build-up of VE between 7–21 days (adults) and 14–27 days (adolescents) after the last dose. No data on VE build-up over time was available for CoronaVac, although serum neutralizing antibody titers peaked at around 2–3 weeks after homologous CoronaVac vaccination^11^. We selected a 7-day delay as our base case because the likelihood value in the inference decreased with longer delay. Nonetheless, we carried out sensitivity analyses assuming VE took effect 14 or 21 days after immunization, which yielded similar VE and waning estimates over time (Fig. 3). Because of the slow rise in cases from late June to the end of July, contemporaneous with the emergence of the BA.4/BA.5 variants (Fig. 1a,b), we performed further sensitivity analyses including only specimens collected by 15 June 2022, which also yielded similar results (Extended Data Fig. 4).

**Fig. 4:**
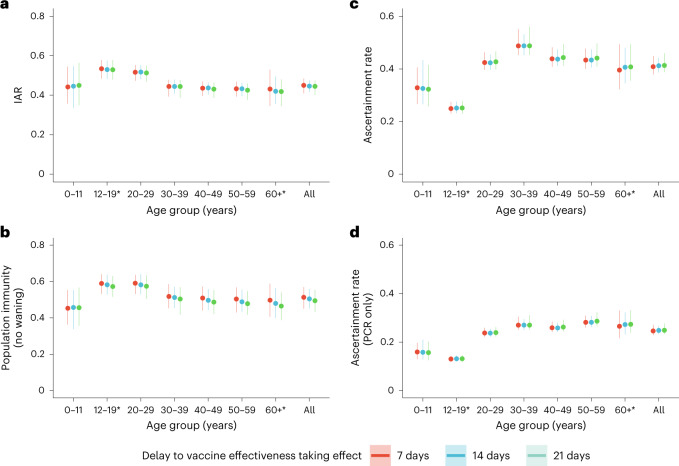
Estimated IAR, population immunity and ascertainment ratio. **a**, Cumulative IAR by 31 July 2022. **b**, Cumulative population immunity from infection and vaccination, assuming no waning of immunity from natural infection, by 31 July 2022. **c**,**d**, Ascertainment rate based on all cumulative confirmed cases (**c**) or cumulative RT–PCR-confirmed cases only (**d**). All analyses were stratified by assumptions on delay to VE taking effect and age group. The dots indicate posterior medians and the lines indicate 95% credible intervals based on the fitted model. *Cumulative IAR, population immunity and ascertainment rate estimates among those aged 12–19 or 60 and above were less accurate because no subjects were between 12 and 17 years old, and few were above 65 years old.

**Fig. 5:**
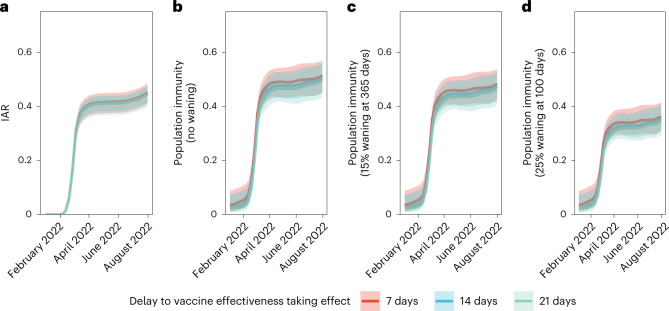
IAR and population immunity over time. **a**, IAR over time. **b**–**d**, Population immunity over time from infection and vaccination, assuming no waning of immunity from natural infection (**b**), immunity from infection wanes by 15% after 365 days (**c**) and immunity from infection wanes by 25% after 100 days (**d**). All analyses were stratified by assumptions on delay to VE taking effect. The lines indicate posterior medians and shaded bars indicate 95% credible intervals based on the fitted model.

**Fig. 6:**
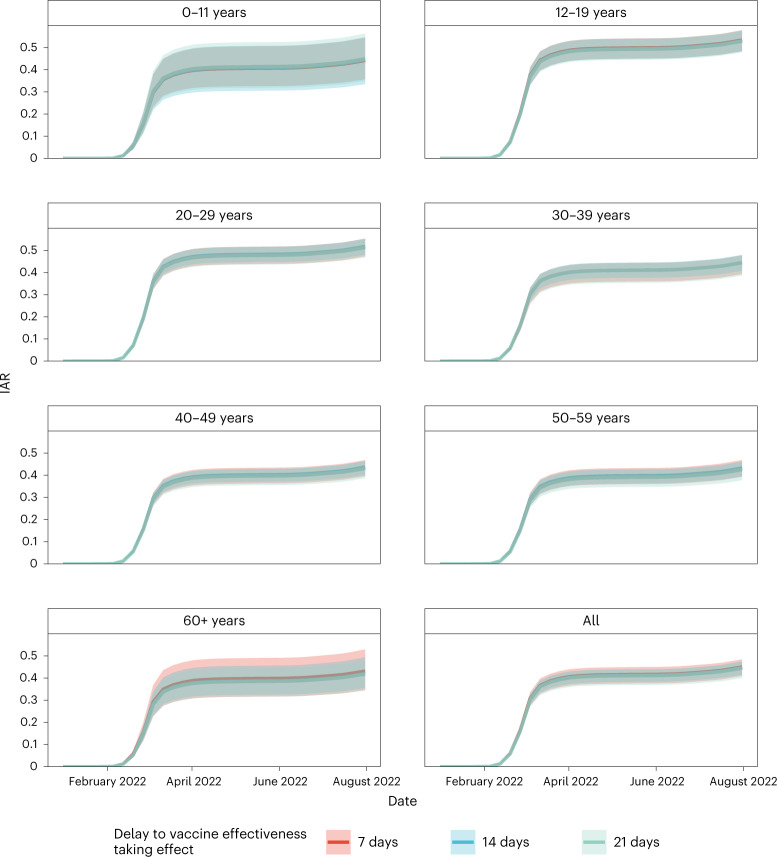
IAR over time by age group. IAR estimates among those aged 12–19 or 60 and above were less accurate because no subjects were between 12 and 17 years old, and few were above 65 years old. The lines indicate posterior medians and shaded bars indicate 95% credible intervals based on the fitted model.

We then estimated age-specific IARs and population immunity over time, together with ascertainment ratios from polymerase chain reaction with reverse transcription (RT–PCR) testing and rapid antigen testing (RAT) using the approach detailed in the Methods. In brief, we first proxied the city-wide FOI via viral load data from wastewater surveillance, adjusted for age effect and calibrated against seropositivity among our unvaccinated study subjects. We then applied our estimates of VE and waning to the (anonymized) vaccination records for every individual in the population provided by the Hong Kong government to derive the probability of infection for each individual until 31 July 2022. Age-specific IARs were then derived by aggregating these probabilities and segmenting into age groups (Figs. 4, 5a and 6). Via this approach, we estimated that SARS-CoV-2 (predominantly Omicron BA.2) infected 45% (41–48%) of the population between 1 January and 31 July 2022. Adolescents and young adults had slightly higher IARs than other age groups. Assuming VE took effect after a 14- or 21-day delay yielded similar IAR estimates. Overall ascertainment ratio was 25% (23–27%) from RT–PCR testing alone, increasing to 41% (38–45%) if augmented with RAT (Fig. 4).

Meanwhile, we defined population immunity as the fraction of the population protected against Omicron BA.2 infection owing to previous infection or vaccination^12,13^, which was equivalent to the relative reduction in the effective reproduction number *R*_*t*_ conferred by natural and vaccine-induced immunity at any given time *t*. Protection conferred by vaccination was assessed based on our estimates of VE and waning. For each individual, we also calculated their probability of infection. We assumed that vaccination-induced and naturally acquired immunity were independent^14^, and that natural infection (with or without previous vaccination) provided perfect protection against reinfection (at least in the short term until the end of our study on 31 July 2022). That is, we did not model the differential protection against reinfection after natural infection between unvaccinated and vaccinated individuals (‘hybrid’ immunity was not modeled).

**Extended Data Fig. 5:**
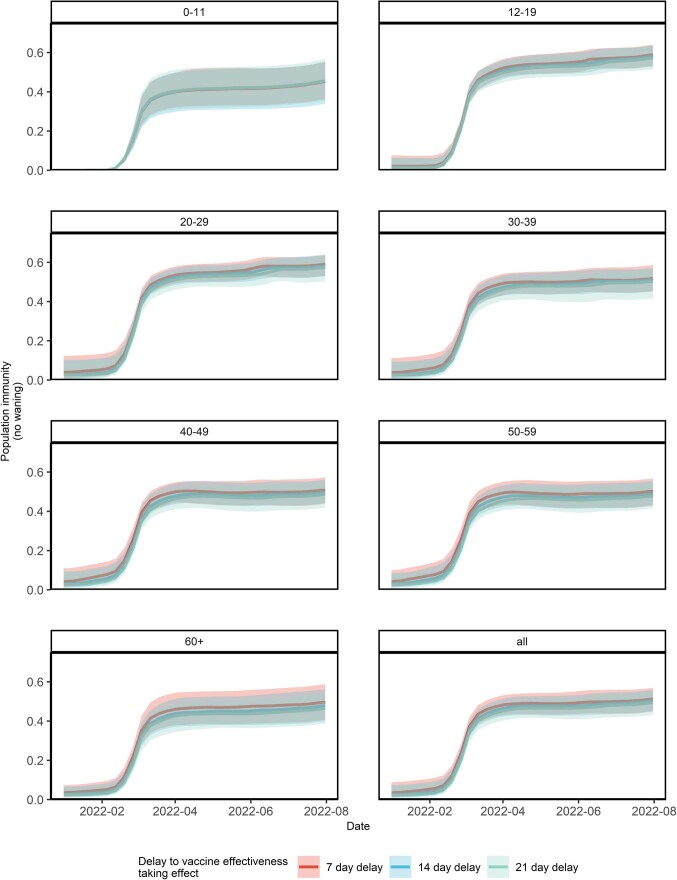
Population immunity over time from vaccination and infection by age group assuming no waning of immunity from infection. Population immunity estimates among those aged 12–19 or aged 60 or above were less accurate as no subjects were between 12 and 17 years old, and few were above 65 years old. The lines indicate posterior medians and shaded bars indicate 95% credible intervals based on the fitted model.

**Extended Data Fig. 6:**
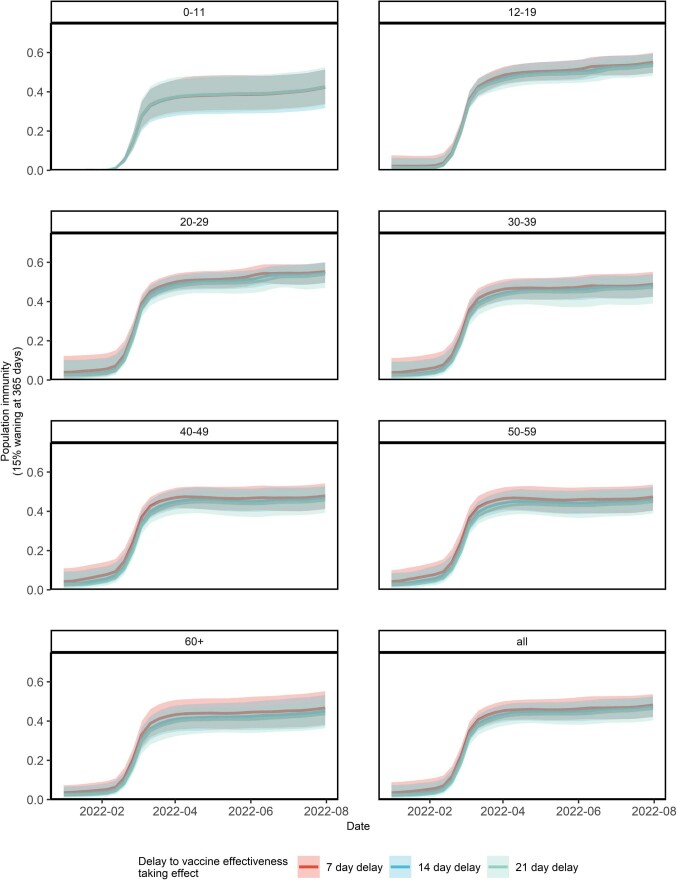
Population immunity over time from vaccination and infection by age group assuming immunity from infection wanes by 15% after 365 days. Population immunity estimates among those aged 12–19 or aged 60 or above were less accurate as no subjects were between 12 and 17 years old, and few were above 65 years old. The lines indicate posterior medians and shaded bars indicate 95% credible intervals based on the fitted model.

**Extended Data Fig. 7:**
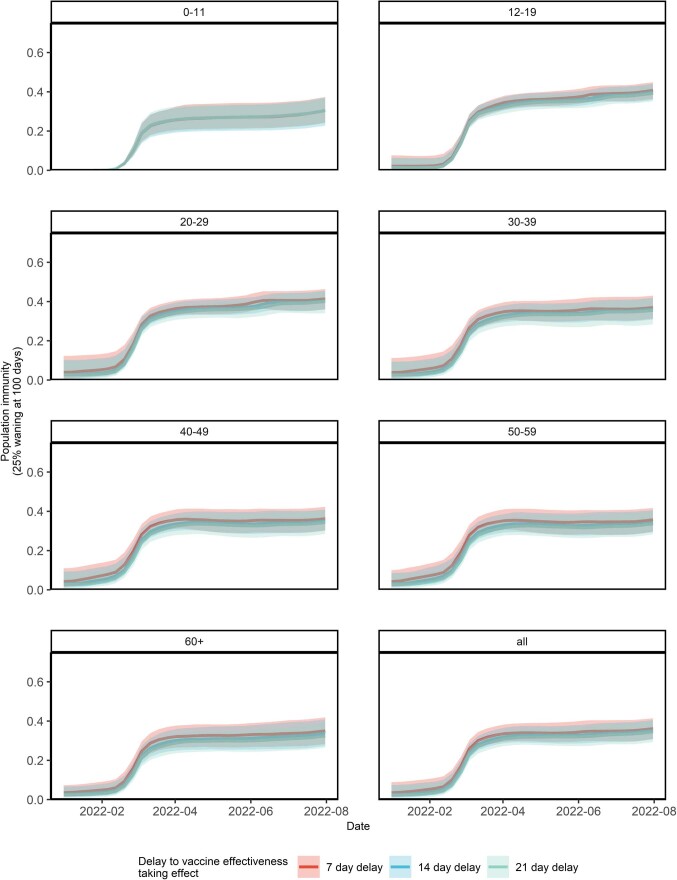
Population immunity over time from vaccination and infection by age group assuming immunity from infection wanes by 25% after 100 days. Population immunity estimates among those aged 12–19 or aged 60 or above were less accurate as no subjects were between 12 and 17 years old, and few were above 65 years old. The lines indicate posterior medians and shaded bars indicate 95% credible intervals based on the fitted model.

We estimated population immunity reached 52% (45–57%) by 31 July 2022. Sensitivity analyses assuming exponentially decaying immunity from natural infection yielded population immunity of 48% (42–54%) or 36% (31–41%) if such naturally acquired immunity decayed to 85% in 365 days^15^ or to 75% in 100 days^16,17^, respectively (Fig. 5 and Extended Data Figs. 5–7). The former estimate of decay was used in medium-term modeling in the UK. The latter, swifter, estimate of decay reflected the emergence and immune escape of the Omicron BA.4/BA.5 variants approximately 3 months after BA.1/BA.2 peaked in Portugal and Qatar—a time frame similar to that experienced in Hong Kong by late July 2022.

## Discussion

Estimating VE against Omicron infection has been challenging in populations that have experienced widespread infection by older variants, owing to difficulties in disentangling the protective effect of vaccine-derived immunity from that of immunity derived from previous infection and ‘hybrid’ immunity. Although the test-negative design has been increasingly used to estimate VE against COVID-19, the robustness of the resulting estimates are typically conditional on symptoms and susceptible to confounding and selection bias (for example, owing to differential healthcare-seeking behavior)^18^. Furthermore, most VE estimates hitherto have estimated protection against symptomatic disease, hospitalization or death but not against all infections including asymptomatic infections. Our estimates of VE against Omicron BA.2 infection are robust against the abovementioned limitations because Hong Kong had negligible infection-derived immunity against any SARS-CoV-2 before to January 2022, and the infection histories of our subjects were individually inferred based on their serological measurements (irrespective of history of symptoms, case confirmation or contact)^19^.

Our Omicron IAR estimates over time were lower than those reported in South Africa (58% in urban areas by April 2022)^20^, Denmark (66% by March 2022)^21^, Navarre, Spain (up to 59% by May 2022)^22^ and British Columbia, Canada (up to 61% by July–August 2022)^23^ likely reflecting the effectiveness of extensive public health and social measures imposed in Hong Kong during the fifth wave, such as a universal mask mandate with high community compliance, closure of all bars, and limits on opening hours and new ventilation requirements in all restaurants^24^, counteracted by the very high density of Hong Kong’s residential dwellings facilitating rapid aerosol transmission between apartments^25^.

Our estimates provide evidence of the short-term effectiveness against Omicron infection of a third or fourth dose of either the mRNA or inactivated vaccine. Slightly higher initial BNT162b2 VE followed by rapid waning has been reported in the literature for symptomatic or RT–PCR-confirmed Omicron BA.2 infection. For example, Chemaitelly et al. reported that effectiveness relative to an unvaccinated reference group against symptomatic infection after the second dose was 51.7% in the first 3 months and waned to ≤10% thereafter, increasing to 43.7% after a booster dose before waning again at similar rate^8^. Meanwhile, Gazit et al. reported a fourth dose of BNT162b2 was 65.1% more effective by the third week against RT–PCR-confirmed Omicron infection relative to a third dose among people aged 60 years or older, declining to 22.0% by the end of 10 weeks^9^, although lower effectiveness was reported in Magen et al.^26^ and Regev-Yochay et al.^27^ also using triple-vaccinated subjects as reference groups. An update to Regev-Yochay et al. reported that the fourth dose of BNT162b2 no longer conferred a statistically significant incremental benefit above the third dose 103–180 days after vaccination^28^, similar to our estimate of VE for BNT162b2 after 100 days.

By contrast, there are very limited data on CoronaVac VE against Omicron infection^29^. Our study provides the first estimate of real-world VE and waning against Omicron infection conferred by three or four doses of CoronaVac. A recent telephone survey in Hong Kong reported three doses of COVID-19 vaccination with either BNT162b2 or CoronaVac provided 52% protection against test-positivity by RT–PCR or RAT relative to unvaccinated individuals, but was unable to account for time since vaccination or for asymptomatic infection^30^. Two South American studies reported 38.2 and 39.8% VE from two doses of CoronaVac against symptomatic Omicron infection in children aged 3–5 and 6–11 years, respectively,^29,31^ also relative to unvaccinated individuals. As a caveat, we distinguish our definition of VE (the reduction in the probability of infection relative to that of an unvaccinated reference group, with infection detected by seropositivity) from the definitions used in the above studies (generally, the reduction in the incidence of infection among a vaccinated and/or boosted intervention arm relative to a reference arm of unvaccinated or less-vaccinated individuals, with infection detected by voluntary RT–PCR or RAT testing). The different definitions might have contributed to the differences between our VE estimates and those of the other studies cited above.

We previously reported markedly reduced serum neutralizing antibody titers against BA.2 among individuals recently vaccinated with three doses of CoronaVac compared with the wild-type virus, with antibody titers below the predicted protective threshold^32^. Thus, our estimate of VE against BA.2 infection elicited by three doses of CoronaVac appears greater than would be expected from neutralizing antibody titers. Indeed, a recent VE study of CoronaVac vaccine using a test-negative design during this same BA.2 epidemic in Hong Kong also observed robust protection from severe disease and death^33^. It is possible that neutralizing antibody titers underestimate protection conferred by whole-virus inactivated vaccines such as CoronaVac, which present multiple viral proteins to the host immune system that may protect via multiple pathways other than neutralizing antibodies, such as T cell immunity and antibody-dependent cytotoxicity^6^.

Nonetheless, we note that the rapid waning of VE over time from even four doses of intramuscular vaccination by monovalent mRNA or inactivated vaccines based on the original Wuhan strain demonstrates the limits of such vaccines in preventing SARS-CoV-2 infection and transmission in the long run. Further, although boosting by BNT162b2 or CoronaVac restores strong protection against hospitalization and death^33–35^, the incremental effectiveness of BNT162b2 boosters against such outcomes waned over the course of 4–6 months^34^ for both adults and the elderly^36^. There are limited data on CoronaVac waning in the Omicron era, though VE against intensive care admission in Malaysia waned considerably among the elderly 3–5 months after the primary series during a period of Delta dominance^37^. If this finding is confirmed in other studies, surge booster vaccination to top-up protection against severe disease and death, particularly among the elderly, remains a key tool in reducing COVID-19’s burden on healthcare systems and mortality before anticipated waves of infections.

Since September 2022, bivalent mRNA vaccines encoding the BA.5 spike protein have become widely available. Early observational evidence on their real-world VE are emerging^38^. Further investigations are necessary to ascertain the VE and waning over time in the midst of our complex SARS-CoV-2 variant and immunity landscape.

Despite the potential of reformulated bivalent boosters in increasing population immunity before upcoming waves of infections, vaccine hesitancy has resulted in very slow uptake of the bivalent boosters since their introduction^39^. The quadruple threat of potentially rapid VE waning, poor vaccine uptake, the possibility for immune imprinting and increasing complex SARS-CoV-2 evolution with the potential for multiple antigenically distant lineages cocirculating simultaneously^40^ may also create formidable challenges in the formulation and rapid deployment of updated bivalent or multivalent vaccines in the near future. There is thus substantial impetus to accelerate the development of mucosal vaccines^41^ and/or universal sarbecovirus vaccines^42^ capable of inducing broad, durable immunity against different variants of SARS-CoV-2 (refs. ^43,44^) to break the chain of transmission and limit the absolute burden of severe disease and long-term sequelae (long covid)^45^ from high-levels of breakthrough COVID-19 infection.

### Limitations

Our study has several important limitations. First, we assumed that the effect of vaccination history on contact patterns and mobility (that is, exposure to the virus), a potential confounder of VE estimates, was negligible. Second, we had no serum samples from individuals aged 12–17 years and only few samples from individuals aged >65 years. As such, our IAR estimates for these age groups were less robust compared with other age groups. We assumed the same IAR among those aged >65 because: (1) 18, 16 and 17% of those aged 60–69, 70–79 and >80 years were confirmed to have COVID-19 during the period 1 January to 31 July 2022 (ref. ^1^); and (2) testing was widely available during the fifth wave and hence the ascertainment ratio was likely to be similar among those aged >65 years. Given the dramatically higher incidence of severe disease and death among the elderly, booster vaccination schemes should continue to prioritize this age cohort, particularly in lower- and middle-income countries with limited vaccine supply.

Third, we were unable to provide estimates of VE against hospitalization, severe disease and death owing to the wide introduction of oral antivirals in both ambulatory and hospital settings on 26 February 2022 in Hong Kong. The antivirals were very effective in further reducing the risk of hospitalization and death among those aged >60 years, including those who were partially vaccinated^46,47^ or, in the case of Nirmatrelvir/Ritonavir (Paxlovid), also those who were fully vaccinated and boosted and had received their most recent dose >20 weeks previously^48^. Any estimate of VE against such outcomes, regardless of study methodology, must therefore account for the use of oral antivirals among its study subjects. Because we did not have access to comprehensive population-level data on oral antiviral usage in Hong Kong (particularly those prescribed in outpatient settings) or complete data matching cases of severe disease and death against their vaccination history and use of oral antivirals, we were unable to derive accurate estimates of VE, which must account for the significant protective effect of oral antivirals.

Fourth, our analysis was primarily based on seroprevalence among blood donors and voluntary child participants recruited from the community in the polio seroepidemiology study who might be healthier and thus not be representative of the general population in terms of their infection history, potentially underestimating seroprevalence. Nonetheless, nations that have relied on blood donors to provide early estimates of seroprevalence have subsequently reported similar estimates among random population samples^49^ or commercial laboratory specimen remnants^50^. In Hong Kong, a separate study of 873 hospital patient plasma specimens detected 43% were anti-N seropositive and 23% were anti-ORF8 seropositive by May 2022 (ref. ^51^). Another separate phylogenetic model of population IAR using GISAID sequences uploaded from multiple laboratories in Hong Kong as input also arrived at upper estimates between 33 and 49% (13–100%) by the week of 16 April 2022, which was similar to our mean estimate of 40% in the same week^52^. These independent estimates provide confidence that our reliance on healthy blood donors and voluntary child participants did not result in a material underestimate of COVID-19 seroprevalence in the general population.

Fifth, the small number of CoronaVac vaccinees in our serosurvey together with the short duration of the fifth wave led to substantial uncertainty in our CoronaVac VE estimates.

Sixth, we were unable to estimate VE conferred by heterologous vaccinations (CoronaVac with BNT162b2 boosters or vice versa) because of the very small number of individuals with such vaccination history (heterologous boosters were not available in Hong Kong until late 2021). When estimating IAR, we assumed that VE for each dose in heterogenous vaccinees equals that of the corresponding dose in homologous vaccinees.

**Extended Data Fig. 8:**
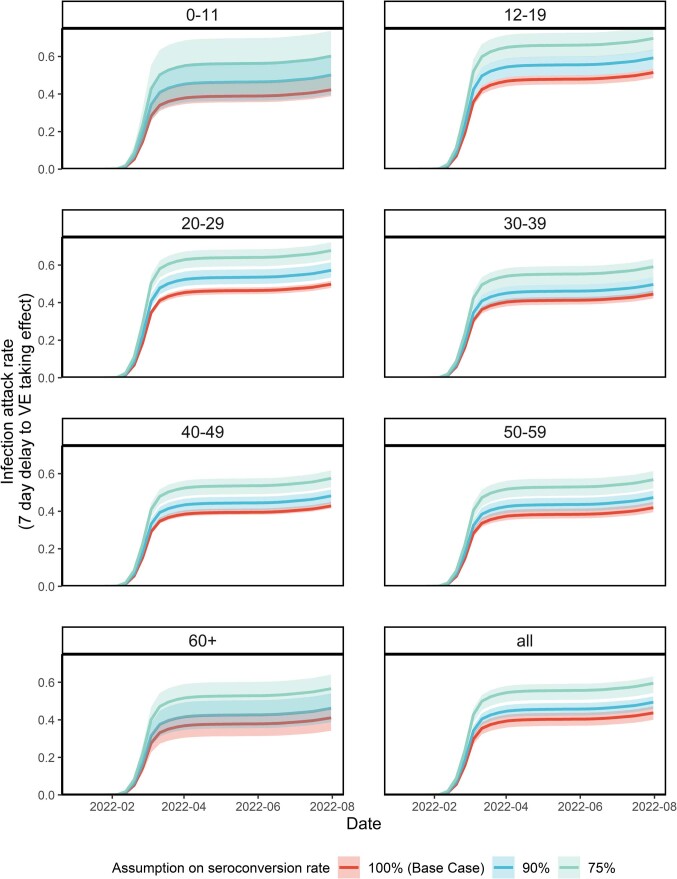
IAR over time by age group and assumptions on seroconversion rate among infected. All figures assumed a 7-day delay to VE taking effect. IAR estimates among those aged 12–19 or aged 60 or above were less accurate as no subjects were between 12 and 17 years old, and few were above 65 years old. The lines indicate posterior medians and shaded bars indicate 95% credible intervals based on the fitted model.

Seventh, because our positive controls comprise only confirmed or self-reported infections, the corresponding seropositivity threshold may not be sufficiently sensitive to detect individuals with asymptomatic or very mild infections, thereby underestimating IAR. Two large pre-Omicron seroepidemiological studies before the availability of vaccines have reported that 5% (ref. ^53^) or 20% (ref. ^54^) of confirmed cases may not seroconvert. We performed sensitivity analyses to estimate the increase in our IAR estimates assuming that 10% or 25% of infected individuals did not seroconvert (that is, corresponding to seroconversion rates of 90 and 75%) (Extended Data Fig. 8). A 90% seroconversion rate would increase our IAR estimate to 50% (46–52%), whereas a 75% seroconversion rate would increase our estimate to 59% (54–63%) by 31 July 2022.

Lastly, because most of our serum samples were collected during and after a period of BA.2 dominance, we were unable to estimate further accelerated VE waning due to the emergence of later variants such as BA.4/BA.5 by late July 2022 in Hong Kong.

In conclusion, our results indicate the short-term effectiveness of booster vaccination using either the mRNA or inactivated vaccine in preventing SARS-CoV-2 Omicron BA.2 infection. As such, surge booster campaigns could be strategically used to rapidly boost population immunity before upcoming waves of infections. The comparatively lower IAR in Hong Kong also highlights the effect of supplementing vaccination campaigns with continued public health and social measures in disease transmission. Nonetheless, in light of the potential for waning of VE, antigenic imprinting and rapid viral evolution, frequently updated studies quantifying the protective effect of repeated booster vaccination, including with the new bivalent COVID-19 vaccines, are necessary for policymakers to develop effective booster vaccination strategies.

## Methods

### Data sources

#### Serosurveys conducted by the study team

As part of a community-based COVID-19 seroepidemiological study, we recruited healthy blood donors by convenience sampling at the five largest blood donation centers (Mongkok, Causeway Bay, Kwun Tong, Tsuen Wan and Shatin) of the Hong Kong Red Cross Blood Transfusion Service from 28 April to 30 July 2022. We also tested serum samples from participants in an independent polio seroepidemiology study targeting children aged 18 months to 10 years from 7 May to 5 August 2022. Child participants in the polio seroepidemiology study were recruited at random from the community via social media, website and word-of-mouth advertising. Blood donors were matched by the Hong Kong Red Cross Blood Transfusion Service and the Hong Kong Department of Health with official vaccination records via unique blood transfusion service donor identification numbers. The records were then anonymized and provided to the study team. Both blood donors and child participants were asked to self-report their vaccination and COVID-19 infection history (as confirmed by RT–PCR testing or RAT pursuant to Hong Kong government guidelines). In cases in which official vaccination records were unavailable (that is, those vaccinated outside Hong Kong), we relied on the donors’ self-reported vaccination history if provided. All child participants self-reported their vaccination and infection history.

Written informed consent was obtained from all participants. Parental consent was obtained for all participants aged <18 years. Further, consent was obtained from the parents of child participants of the polio seroepidemiology survey to test collected sera for antibodies and/or biomarkers specific to a panel of pathogens other than polio, including but not limited to SARS-CoV-2, seasonal influenza, respiratory syncytial virus, human metapneumovirus, adenovirus, rhinovirus, enterovirus and human parainfluenza virus. Blood donor participants received no compensation for their participation. Child participants received compensation of HK $1,000 for participating in the polio seroepidemiology study. Ethical approval for this study and the polio seroepidemiology study (including the use of samples collected therein for antibody or biomarker testing against nonpolio pathogens) were obtained from the Institutional Review Board of the Hospital Authority Hong Kong West Cluster/University of Hong Kong (IRB No. UW 20–132 and IRB No. UW 21–196, respectively).

#### Vaccination records, confirmed cases and sewage surveillance data provided by the Hong Kong government

Official vaccination records in Hong Kong are maintained by the Hong Kong Department of Health^55^. Anonymous data on every vaccination up to 31 July 2022, including the date of each dose, type of vaccine (BNT162b2 or CoronaVac) used and vaccinee year-of-birth, were compiled by the Department of Health and provided to us by the Hong Kong Office of the Government Chief Information Officer. Age data on all confirmed SARS-CoV-2 cases were provided by the Centre for Health Protection. Daily per capita 2-day running geometric mean SARS-CoV-2 viral load data (in copies of SARS-CoV-2 RNA l^−1^) obtained from city-wide COVID-19 wastewater surveillance up to 31 July 2022 were provided by the Hong Kong Environmental Protection Department. The 2022 projected mid-year population in each age cohort was obtained from the Hong Kong Census and Statistics Department^56^.

### Laboratory methods

We developed two in-house ELISA assays that detected IgG antibodies to N-CTD and ORF8 (ref. ^57^) of SARS-CoV-2, respectively, modifying the methodology reported in Mok et al.^6^ and Hachim et al^7^. The ORF8 assay was developed specifically for detecting past Omicron BA.2 infections in CoronaVac vaccinees because most of them were N-CTD-seropositive owing to the immune response that CoronaVac elicits against the N protein. The ELISA assays as previously described^6,7^ were optimized and validated. In brief, 96-well ELISA plates (Nunc MaxiSorp, Thermo Fisher Scientific) were coated overnight with 40 ng well^−1^ of purified recombinant N-CTD protein in PBS buffer for the N-CTD protein ELISA assay or 30 ng well^−1^ of purified recombinant ORF8 protein in PBS buffer for the ORF8 ELISA assay. The plates were then blocked by 100 μl of Chonblock blocking buffer (Chondrex) per well and incubated at room temperature for 2 h. Each serum sample was tested at a dilution of 1:100 in Chonblock blocking buffer in duplicate. The serum dilutions were added and incubated for 2 h at 37 °C. After extensive washing with PBS containing 0.2% Tween 20, horseradish peroxidase-conjugated goat anti-human IgG (1:5,000; GE Healthcare) was added and incubated for 1 h at 37 °C. The ELISA plates were then washed again with PBS containing 0.2% Tween 20. Subsequently, 100 μl of horseradish peroxidase substrate (Ncm TMB One; New Cell and Molecular Biotech) was added into each well. After 15 min incubation, the reaction was stopped by adding 50 μl of 2 M H_2_SO_4_ solution and analyzed on a microplate reader at 450 nm wavelength. Positive and negative controls were included in each run.

This resulted in cutoffs of 0.2583 and 0.33 optical density (OD) for N-CTD and ORF8, respectively. The assays and cutoffs were validated against pre-pandemic blood donor samples, blood samples from homologous BNT162b2- or CoronaVac-vaccinated individuals collected during periods of minimal community transmission in 2020 and 2021, blood samples collected from RT–PCR-confirmed SARS-CoV-2 convalescent individuals in 2020 and 2021, and samples from blood donors in the current study with self-reported infection history. See Extended Data Fig. 3 for details on control groups and assay performance (sensitivity, specificity and receiver operating curves). We set the ELISA cutoffs to approximately maximize the sum of sensitivity and specificity, which were in turn estimated via bootstrapping 2,000 samples using the pROC R package^58^, with specificity set not lower than 90%.

We did not account for waning of N-CTD and ORF8 antibody response. Nonetheless, we previously reported that N- and ORF8-specific antibody responses were well maintained for at least 100 days postinfection^7^. This is on par with the time elapsed between infection (for example, the fifth wave peaked in early March 2022) and the time of sample collection (between late April and July 2022) for our serosurvey subjects.

### Statistical methods

#### Statistical inference of VE

Let ${\mathrm{VE}}_{v,j}\left( u \right)$ be the VE of vaccine type *v* (B for BNT162b2 and C for CoronaVac) against infection *u* days after the *j*th dose in a homologous series has taken effect. For each vaccine type *v*, we assumed: (1) the first dose provided no protection against infection, that is ${\mathrm{VE}}_{v,1}\left( u \right) = 0$ (ref. ^59^); (2) ${\mathrm{VE}}_{v,j}\left( 0 \right)$ depended on the number but not the time of previous doses; (3) VE waned exponentially at a constant rate *λ*_*v*_ after each dose^5,60–62^, that is ${\mathrm{VE}}_{v,j}\left( u \right) = {\mathrm{VE}}_{v,j}\left( 0 \right) \times \exp \left( { - \lambda _vu} \right)$; and (4) ${\mathrm{VE}}_{v,j}\left( 0 \right)$ increased with each successive dose in a homologous series, that is ${\mathrm{VE}}_{v,j + 1}\left( 0 \right) > {\mathrm{VE}}_{v,j}\left( 0 \right)$ (refs. ^26,63–65^). We also assumed that the initial VE of two-dose BNT162b2 was not inferior to that of two-dose CoronaVac, that is ${\mathrm{VE}}_{{\mathrm{B}},2}\left( 0 \right) \ge {\mathrm{VE}}_{{\mathrm{C}},2}\left( 0 \right)$ (the latter was not statistically identifiable otherwise).

Let time 0 be 1 January 2021. We assumed that the FOI at time *t* was proportional to the viral load per capita from city-wide sewage. Specifically, given an individual aged *a* with vaccination history *H* who remained uninfected at time *t*, her FOI at that time was

$$\lambda \left( t \right) = \gamma \times f\left( a \right) \times {\mathrm{VL}}\left( t \right) \times \left( {1 - {\mathrm{VE}}_{v,n\left( t \right)}\left( {t - T_{n(t)}} \right) }\right)$$

where:

1. *f*(*a*) was the effect of age on FOI with *f*(35) = 1 (those aged 35 years were the reference group). We assumed that: (1) *f*(*a*) was a piecewise cubic Hermite interpolating polynomial function for 10 ≤ *a* ≤ 65 with knots at 10, 18, 35, 50 and 65 years; and (2) *f*(*a*) = *f*(10) for *a* < 10 and *f*(*a*) = *f*(65) for *a* > 65.
2. VL(*t*) was the two-day running geometric mean viral load per capita from city-wide sewage.
3. *n*(*t*) was the total number of doses of vaccine type *v* that the individual had received up to time *t* and *T*_*n*(*t*)_ was the time at which the most recent dose took effect.
4. *γ* was a scaling factor (subject to statistical inference; Supplementary Table 1).

The probability that this individual was infected between time 0 and *t* was $p_{{\mathrm{infection}}}\left( {t|a,H} \right) = 1 - \exp\left( { - {\int}_0^t {\lambda \left( u \right){\mathrm{d}}u} } \right)$. If tested at the time *t*, this individual would be seropositive with probability

$$\begin{array}{l}p_{{\mathrm{seropositive}}}\left( {t|a,H} \right) = q_{{\mathrm{sens}},v} \times p_{{\mathrm{infection}}}\left( {t|a,H} \right)+ \left( {1 - q_{{\mathrm{spec}},v}} \right)\\ \times \left( {1 - p_{{\mathrm{infection}}}\left( {t|a,H} \right)} \right)\end{array}$$

where $q_{{\mathrm{sens}},v}$ and $q_{{\mathrm{spec}},v}$ were the sensitivity and specificity of the serological assay that we used to infer previous Omicron infections for individuals vaccinated with vaccine type *v*.

Let **θ** denote the set of model parameters subject to statistical inference (Supplementary Table 1). Let **D** denote the data available for inferring **θ** which comprised:

1. The age, vaccination history and time of serum collection of each subject *i* in the serosurvey. These data were used to calculate the probability of seropositivity of the serum sample collected from subject *i* ($p_{{\mathrm{seropositive}},i}$) via the abovementioned model.
2. The observed seropositivity of the serum sample for each subject *i* in the serosurvey (*τ*_*i*_ = 1 if seropositive and *τ*_*i*_ = 0 otherwise).
3. The number of positive and negative controls for estimating the sensitivity and specificity of our in-house ELISA assays among individuals with different vaccination history ($n_{{\mathrm{sens}},v}$ and $n_{{\mathrm{spec}},v}$) and the respective number of seropositive samples ($y_{{\mathrm{sens}},v}$ and $y_{{\mathrm{spec}},v}$). See Extended Data Fig. 3 for details.

We used the following likelihood function to infer **θ** from **D**:

$$\begin{array}{l}L\left( {{\mathbf{\uptheta }}|{{{\mathbf{D}}}}} \right) = \mathop {\prod}\limits_{i \in {\mathrm{Serosurvey}}} {{\mathrm{Bernoulli}}\left( {\tau _i|p_{{\mathrm{seropositive}},i}} \right)} \\ \times \mathop {\prod}\limits_{v \in \left\{ {U,B,C} \right\}} {{\mathrm{Binomial}}\left( {y_{{\mathrm{sens}},v}|n_{{\mathrm{sens}},v},q_{{\mathrm{sens}},v}} \right)} \\ \times \mathop {\prod}\limits_{v \in \left\{ {U,B,C} \right\}} {{\mathrm{Binomial}}\left( {y_{{\mathrm{spec}},v}|n_{{\mathrm{spec}},v},1 - q_{{\mathrm{spec}},v}} \right)} \end{array}$$

where ${\mathrm{Bernoulli}}\left( { \cdot |p} \right)$ was the Bernoulli pdf with parameter *p*, ${\mathrm{Binomial}}\left( { \cdot |n,q} \right)$ was the Binomial pdf with *n* trials and success probability *q*. The statistical inference was performed in a Bayesian framework with noninformative (flat) priors using Markov Chain Monte Carlo with Gibbs sampling. We used *P*(**θ**) to denote the posterior distribution of **θ** obtained from fitting the model to the data **D**.

#### Estimating IAR and population immunity

We randomly drew 300 samples of **θ** from *P*(**θ**). For each sample of **θ** drawn, we calculated $p_{{\mathrm{infection}},i}(t)$ (cumulative probability of infection) and ${\mathrm{RE}}_i(t) + \left( {1 - p_{{\mathrm{infection}},i}(t)} \right) \times {\mathrm{VE}}_{v,j}\left( t \right)$ (expected immunity) for each individual in the general population given their vaccination record (as done for our serosurvey subjects) on days *t* at weekly intervals between 1 January and 31 July 2022. The protection conferred by previous infection against reinfection on day *t* is:

$${\mathrm{RE}}_i\left( t \right) = {\int}_0^t {p_{{\mathrm{infection}},i}^\prime \left( \tau \right) \times {{{\mathrm{exp}}}}\left( { - \kappa \left( {t - \tau } \right)} \right){\mathrm{d}}\tau }$$

where:

1. $p_{{\mathrm{infection}},i}^\prime \left( \tau \right) = \lambda \left( \tau \right)\exp\left( { - {\int}_0^\tau {\lambda \left( u \right){\mathrm{d}}u} } \right)$ is the probability of getting infected at time *τ*
2. *κ* is the waning rate of immunity conferred by previous infection.

Three *κ* scenarios were considered: (1) *κ* = 0 (base case, no waning); (2) $\kappa = - \log \left( {0.85} \right)/365$ (corresponding to the ‘high waning’ scenario of 15% in one year in Barnard et al.^15^); and (3) $\kappa = - {{{\mathrm{log}}}}(0.75)/100$ (corresponding to a 25% loss of protection within 100 days as observed in Portugal^16^ and Qatar^17^ upon BA.4/BA.5 emergence).

Posterior medians and 95% credible intervals of age-specific IARs and population immunity were compiled accordingly.

For individuals with heterologous CoronaVac and BNT162b2 vaccinations, we assumed VE for each dose was the same as that of the corresponding type and dose in a homologous series. We substituted missing records for intervening or preceding doses with the vaccine type of the next recorded dose, with a 90-day gap between the third and fourth doses, 180-day gap between the second and third doses or a 14-day gap between the first and second doses as per Hong Kong government recommendations before 31 May 2022. We derived the number of unvaccinated individuals in each age cohort based on the 2022 predicted mid-year population per the Census and Statistics Department^56^.

Lastly, we calculated the median and 95% confidence intervals of IARs, population immunity and ascertainment ratios by age group. We further performed sensitivity analyses incorporating the posterior distribution corresponding to 2-week (14 days) or a 3-week (21 days) delay for VE to take effect after each dose (Figs. 3–6 and Extended Data Figs. 5–7).

All analyses were performed using MATLAB 2022a with the Parallel Computing and Econometrics toolboxes and R v.4.2.1, with the tidyverse (v.1.3.2), pROC (v.1.18.0), cowplot (v.1.1.1) and janitor (v.2.1.0) packages.

### Reporting summary

Further information on research design is available in the Nature Portfolio Reporting Summary linked to this article.

## Online content

Any methods, additional references, Nature Portfolio reporting summaries, source data, extended data, supplementary information, acknowledgements, peer review information; details of author contributions and competing interests; and statements of data and code availability are available at 10.1038/s41591-023-02219-5.

### Supplementary information

Supplementary Information Supplementary Table 1: Parameters subject to statistical inference.

Reporting Summary

## Data Availability

The anonymized vaccination record data were compiled by the Office of the Government Chief Information Officer (OGCIO) (enquiry@ogcio.gov.hk) and the Department of Health (enquiries@dh.gov.hk), The Government of Hong Kong Special Administrative Region (HKSAR). Age data for confirmed cases were compiled by the Centre for Health Protection (enquiries@dh.gov.hk). Data on viral load from sewage surveillance were compiled by the Environmental Protection Department, The Government of HKSAR (enquiry@epd.gov.hk). The aforementioned data could not be shared due to confidentiality undertakings to the above-named agencies. Interested parties could contact these agencies for access to these data. Request for access to anonymized serology output data may be directed to the corresponding author. However, as this data is matched to vaccination records covered by the aforementioned confidentiality undertaking, access is also subject to preapproval by the above-named agencies of The Government of HKSAR. Outputs of our analysis and source data for Figs. 3–6 and Extended Data Figs. 1 and 4–8 are accessible at https://github.com/jonathanjlau-hku/hkserosurvey2022.

## Code availability

All code files are accessible at https://github.com/jonathanjlau-hku/hkserosurvey2022.
