# Author Correction: Real-world COVID-19 vaccine effectiveness against the Omicron BA.2 variant in a SARS-CoV-2 infection-naive population

**DOI:** 10.1038/s41591-023-02648-2

**Published:** 2023-10-24

**Authors:** Jonathan J. Lau, Samuel M. S. Cheng, Kathy Leung, Cheuk Kwong Lee, Asmaa Hachim, Leo C. H. Tsang, Kenny W. H. Yam, Sara Chaothai, Kelvin K. H. Kwan, Zacary Y. H. Chai, Tiffany H. K. Lo, Masashi Mori, Chao Wu, Sophie A. Valkenburg, Gaya K. Amarasinghe, Eric H. Y. Lau, David S. C. Hui, Gabriel M. Leung, Malik Peiris, Joseph T. Wu

**Affiliations:** 1 WHO Collaborating Centre for Infectious Disease Epidemiology and Control, School of Public Health, Li Ka Shing Faculty of Medicine, The University of Hong Kong, Hong Kong SAR, China; 2 Laboratory of Data Discovery for Health (D24H), Hong Kong SAR, China; 3 School of Public Health, Li Ka Shing Faculty of Medicine, The University of Hong Kong, Hong Kong SAR, China; 4 The University of Hong Kong – Shenzhen Hospital, Shenzhen, China; 5 Hong Kong Red Cross Blood Transfusion Service, Hong Kong SAR, People’s Republic of China; 6 HKU-Pasteur Research Pole, Li Ka Shing Faculty of Medicine, The University of Hong Kong, Hong Kong SAR, China; 7 Research Institute for Bioresources and Biotechnology, Ishikawa Prefectural University, Nonoichi, Japan; 8 Department of Pathology and Immunology, Washington University School of Medicine at St. Louis, St. Louis, MO USA; 9 Department of Microbiology and Immunology, Peter Doherty Institute for Infection and Immunity, University of Melbourne, Melbourne, Victoria Australia; 10 Department of Medicine and Therapeutics and Stanley Ho Centre for Emerging Infectious Diseases, Faculty of Medicine, Chinese University of Hong Kong, Hong Kong SAR, China; 11 Centre for Immunology and Infection, Hong Kong SAR, China

**Keywords:** Viral infection, Epidemiology, SARS-CoV-2, Vaccines

Correction to: *Nature Medicine* 10.1038/s41591-023-02219-5. Published online 18 January 2023.

In the version of the article initially published, there was a typographical error in the force of infection (FOI) formula under the second paragraph of the “Statistical Methods” section, originally reading $\lambda \left(t\right)=\gamma \times f(a)\times {VL}(t)\times {{VE}}_{v,n\left(t\right)}\left(t-{T}_{n\left(t\right)}\right)$. The formula has been corrected to read $\lambda \left(t\right)=\gamma \times f(a)\times {VL}(t)\times \left(1-{{VE}}_{v,n\left(t\right)}\left(t-{T}_{n\left(t\right)}\right)\right)$ in the HTML and PDF versions of the article. The results of the article were not affected by this error.

