# Supplementary material for: Real-world COVID-19 vaccine effectiveness against the Omicron BA.2 variant in a SARS-CoV-2 infection-naive population
Source: Nat Med. 2023 Jan 18;29(2):348–57. doi: 10.1038/s41591-023-02219-5 (PMC9941049; doi:10.1038/s41591-023-02219-5)
Supplement: Supplementary file 1 — Supplementary Table 1: Parameters subject to statistical inference. [file 41591_2023_2219_MOESM1_ESM.pdf]

nature medicine

Article

https://doi.org/10.1038/s41591-023-02219-5

Real-world COVID-19 vaccine effectiveness against the Omicron BA.2 variant in a SARS-CoV-2 infection-naive population

In the format provided by the authors and unedited

Supplementary Table 1. Parameters subject to statistical inference.

**Parameters**	**Description**
$VE_{v,j}(0)$ for $v \in \{B, C\}, j \in \{2,3,4\}$	Initial vaccine effectiveness conferred by each successive dose of vaccine
λ_v for $v = \{B, C\}$	Waning rate of VE
γ	The scaling factor that related FOI to sewage viral load
$f(10), f(18), f(50)$ and $f(65)$	The effect of age on FOI for age 10, 18, 50 and 65 years
$q_{sens,v}$ and $q_{spec,v}$ for $v \in \{U, B, C\}$	Sensitivity and specificity of serological assays for detecting recent Omicron infection among unvaccinated individuals (*U*) and those vaccinated with BNT162b2 (*B*) or Coronavac (*C*)
