# Supplementary material for: Real-world COVID-19 vaccine effectiveness against the Omicron BA.2 variant in a SARS-CoV-2 infection-naive population
Source: Nat Med. 2023 Jan 18;29(2):348–57. doi: 10.1038/s41591-023-02219-5 (PMC9941049; doi:10.1038/s41591-023-02219-5)
Supplement: Supplementary file 2 — Reporting Summary [file 41591_2023_2219_MOESM2_ESM.pdf]

nature portfolio

Corresponding author(s): Joseph T. Wu

Last updated by author(s): 01/13/2023

Reporting Summary

Nature Portfolio wishes to improve the reproducibility of the work that we publish. This form provides structure for consistency and transparency in reporting. For further information on Nature Portfolio policies, see our Editorial Policies and the Editorial Policy Checklist.

Statistics

For all statistical analyses, confirm that the following items are present in the figure legend, table legend, main text, or Methods section.

n/a	Confirmed	
☐	☒	The exact sample size (n) for each experimental group/condition, given as a discrete number and unit of measurement
☐	☒	A statement on whether measurements were taken from distinct samples or whether the same sample was measured repeatedly
☒	☐	The statistical test(s) used AND whether they are one- or two-sided<br>*Only common tests should be described solely by name; describe more complex techniques in the Methods section.*
☐	☒	A description of all covariates tested
☐	☒	A description of any assumptions or corrections, such as tests of normality and adjustment for multiple comparisons
☐	☒	A full description of the statistical parameters including central tendency (e.g. means) or other basic estimates (e.g. regression coefficient) AND variation (e.g. standard deviation) or associated estimates of uncertainty (e.g. confidence intervals)
☒	☐	For null hypothesis testing, the test statistic (e.g. F, t, r) with confidence intervals, effect sizes, degrees of freedom and P value noted<br>*Give P values as exact values whenever suitable.*
☐	☒	For Bayesian analysis, information on the choice of priors and Markov chain Monte Carlo settings
☒	☐	For hierarchical and complex designs, identification of the appropriate level for tests and full reporting of outcomes
☒	☐	Estimates of effect sizes (e.g. Cohen's d, Pearson's r), indicating how they were calculated

Our web collection on statistics for biologists contains articles on many of the points above.

Software and code

Policy information about availability of computer code

Data collection	MATLAB 2022a with the Parallel Computing and Econometrics toolboxes and R 4.2.1, with the tidyverse (v.1.3.2), pROC (v.1.18.0), cowplot (v.1.1.1) and janitor (v.2.1.0) packages.<br><br>Code is available at: https://github.com/jonathanjlau-hku/hkserosurvey2022
Data analysis	MATLAB 2022a with the Parallel Computing and Econometrics toolboxes and R 4.2.1, with the tidyverse (v.1.3.2), pROC (v.1.18.0), cowplot (v.1.1.1) and janitor (v.2.1.0) packages.<br><br>Code is available at: https://github.com/jonathanjlau-hku/hkserosurvey2022

For manuscripts utilizing custom algorithms or software that are central to the research but not yet described in published literature, software must be made available to editors and reviewers. We strongly encourage code deposition in a community repository (e.g. GitHub). See the Nature Portfolio guidelines for submitting code & software for further information.

Data

Policy information about availability of data

All manuscripts must include a data availability statement. This statement should provide the following information, where applicable:

- Accession codes, unique identifiers, or web links for publicly available datasets
- A description of any restrictions on data availability
- For clinical datasets or third party data, please ensure that the statement adheres to our policy

The anonymized vaccination record data were compiled by the Office of the Government Chief Information Officer (OGCIO) (enquiry@ogcio.gov.hk) and the Department of Health (enquiries@dh.gov.hk), The Government of Hong Kong Special Administrative Region (HKSAR). Age data for confirmed cases were compiled by the Centre for Health Protection (enquiry_chpweb@dh.gov.hk). Data on viral load from sewage surveillance were compiled by the Environmental Protection Department, The Government of HKSAR (enquiry@epd.gov.hk). The aforementioned data could not be shared due to confidentiality undertakings to the above-named agencies. Interested parties could contact these agencies for access to these data.

Request for access to anonymised serology output data may be directed to the corresponding author. However, as this data is matched to vaccination records covered by the aforementioned confidentiality undertaking, access is also subject to pre-approval by the above-named agencies of The Government of the HKSAR.

Outputs of our analysis and source data for Figs. 3-6 and Extended Data Figs. 1 and 4-8 are accessible at https://github.com/jonathanjlau-hku/hkserosurvey2022.

Human research participants

Policy information about studies involving human research participants and Sex and Gender in Research.

Reporting on sex and gender	Sex information was self-reported by participants. However, sex and gender were not considered in the study design. Summary-level sex information was reported in Table 1 of the manuscript.
Population characteristics	Age and COVID-19 vaccination history were obtained from the Department of Health and the Office of the Government Chief Information Officer (OGCIO), the Government of the HKSAR, or self-reported by participants. SARS-CoV-2 infection history was self-reported by participants. Summary-level population characteristics were reported in Table 1 of the manuscript.
Recruitment	As part of a community-based COVID-19 sero-epidemiological study, we recruited healthy blood donors by convenience sampling at the five largest blood donation centres (Mongkok, Causeway, Kwun Tong, Tsuen Wan and Shatin) of the Hong Kong Red Cross Blood Transfusion Service from 28 April 2022 to 30 July 2022. We also tested serum samples from participants of an independent polio sero-epidemiology study targeting children aged 18 months to 10 years from 7 May to 5 August 2022. Participants in the polio sero-epidemiology study were recruited at random from the community via social media, website and word of mouth advertising.<br><br>We note that blood donors and voluntary children participants recruited from the community in the polio sero-epidemiology study might be healthier and thus not representative of the general population in terms of their infection history, potentially underestimating seroprevalence. However, two other independent studies in Hong Kong (refs 51 and 52 on our manuscript) have produced similar or lower levels of, respectively, seropositivity and infection attack rate versus our work, providing confidence that we did not materially underestimate COVID-19 seroprevalence in the general population.
Ethics oversight	Ethical approvals for this study and the polio sero-epidemiology study were obtained from the Institutional Review Board of the Hospital Authority Hong Kong West Cluster / University of Hong Kong (IRB No. UW 20-132 and IRB No. UW 21-196, respectively).

Note that full information on the approval of the study protocol must also be provided in the manuscript.

Field-specific reporting

Please select the one below that is the best fit for your research. If you are not sure, read the appropriate sections before making your selection.

☒ Life sciences ☐ Behavioural & social sciences ☐ Ecological, evolutionary & environmental sciences

For a reference copy of the document with all sections, see nature.com/documents/nr-reporting-summary-flat.pdf

Life sciences study design

All studies must disclose on these points even when the disclosure is negative.

Sample size	Our serosurvey subjects included: (i) 5,173 healthy adult blood donors recruited from the Hong Kong Red Cross Blood Transfusion Service between 28 April and 30 July 2022; and (ii) 137 children aged 18 months to 11 years randomly recruited from the community to participate in an independent polio sero-epidemiology study.

	Assuming that our serological assays could reliably infer previous infection in both vaccinated and unvaccinated individuals, we calculated that a cross-sectional sample of 100 subjects (for each age group) would allow us to empirically measure the infection attack rate with less than 10% error with probability 0.95, respectively. Our actual sample size exceeded this threshold in all age groups.
Data exclusions	67 participants with non-BNT162b2 or non-CoronaVac, or undetermined, vaccination history and 1 participant with undetermined age
Replication	Interested parties could contact the Office of the Government Chief Information Officer (OGCIO), the Department of Health, the Centre for Health Protection and the Environmental Protection Department, all of the Government of the HKSAR, for access to anonymised vaccination data, age data on confirmed cases and viral load data from sewage surveillance, respectively.<br><br>Please note that the above data, which forms the core of our analysis, is necessary for our full results to be replicated.
Randomization	Randomisation was not relevant to our study as study subjects were not pre-allocated to the intervention studied (vaccine status, type and dose) prior to blood donation and/or recruitment.
Blinding	As study subjects were not pre-allocated to the intervention studied (vaccine status, type and dose) prior to blood donation and recruitment, blinding was not possible.

Reporting for specific materials, systems and methods

We require information from authors about some types of materials, experimental systems and methods used in many studies. Here, indicate whether each material, system or method listed is relevant to your study. If you are not sure if a list item applies to your research, read the appropriate section before selecting a response.

Materials & experimental systems

n/a	Involved in the study
☐	☒ Antibodies
☒	☐ Eukaryotic cell lines
☒	☐ Palaeontology and archaeology
☒	☐ Animals and other organisms
☒	☐ Clinical data
☒	☐ Dual use research of concern

Methods

n/a	Involved in the study
☒	☐ ChIP-seq
☒	☐ Flow cytometry
☒	☐ MRI-based neuroimaging

Antibodies

Antibodies used	Goat anti-Human IgG (H+L) Secondary Antibody, HRP: ThermoFisher Scientific. Catalog # 31410
Validation	-Goat anti-Human IgG (H+L) Secondary Antibody, HRP was validated by the manufacturer using Western blot (non-reducing). A 150 kDa band which correspond to Human IgG was observed in IM-9, ARH-77 and IM-9, ARH-77 conditioned medium8203(CM) but not in low expression Raji, Molt-4, Jurkat CM.<br><br>- Corresponding bands of Human IgG Heavy and Light Chain with ~55kDa and ~25 kDa in size respectively was observed in Human IgG but not in IgA, IgE, IgM, Mouse IgG, Mouse IgM and Rabbit IgG.
